# Microorganisms as a Potential Source of Molecules to Control Trypanosomatid Diseases

**DOI:** 10.3390/molecules26051388

**Published:** 2021-03-04

**Authors:** Manuel Jesús Chan-Bacab, María Manuela Reyes-Estebanez, Juan Carlos Camacho-Chab, Benjamín Otto Ortega-Morales

**Affiliations:** Departamento de Microbiología Ambiental y Biotecnología, Universidad Autónoma de Campeche, Av. Agustín Melgar s/n, Campeche 24039, Mexico; mamreyes@uacam.mx (M.M.R.-E.); juanccam@uacam.mx (J.C.C.-C.); beortega@uacam.mx (B.O.O.-M.)

**Keywords:** microbial metabolites, antitrypanosomatid agents, leishmaniasis, trypanosomiasis

## Abstract

Trypanosomatids are the causative agents of leishmaniasis and trypanosomiasis, which affect about 20 million people in the world’s poorest countries, leading to 95,000 deaths per year. They are often associated with malnutrition, weak immune systems, low quality housing, and population migration. They are generally recognized as neglected tropical diseases. New drugs against these parasitic protozoa are urgently needed to counteract drug resistance, toxicity, and the high cost of commercially available drugs. Microbial bioprospecting for new molecules may play a crucial role in developing a new generation of antiparasitic drugs. This article reviews the current state of the available literature on chemically defined metabolites of microbial origin that have demonstrated antitrypanosomatid activity. In this review, bacterial and fungal metabolites are presented; they originate from a range of microorganisms, including cyanobacteria, heterotrophic bacteria, and filamentous fungi. We hope to provide a useful overview for future research to identify hits that may become the lead compounds needed to accelerate the discovery of new drugs against trypanosomatids.

## 1. Introduction

Infectious tropical diseases constitute a problem for many human beings that inhabit tropical areas of our planet. Affecting people who live in developing countries, these neglected diseases are caused by viruses, protozoa, helminths, and bacteria, which generate different symptoms and may often lead to death [1]. Parasitic diseases have an overwhelming impact on public health, and their geographical distribution favors climates that allow vector persistence for transmission. Vector control is possible, eradication is probably not possible, and vaccine development has so far been unsuccessful, as parasites are experts at evading or deregulating the human immune system [2,3].

Trypanosomatids are flagellated unicellular protozoan parasites belonging to the order Kinetoplastida, family Trypanosomatidae. They are characterized by a single, large mitochondrion that extends through most of these organisms’ bodies, and whose DNA creates a unique and elaborate structure called the kinetoplast, located near the flagellar basal body [4,5]. Several species within the Trypanosomatidae family are responsible for the severe but largely neglected diseases of humans and domestic animals, such as *Leishmania* and *Trypanosoma*, the causative agents of leishmaniasis, American trypanosomiasis (Chagas disease), and Human African trypanosomiasis [6,7].

Although leishmaniasis and trypanosomiasis are targeted for control or eradication by the World Health Organization Division of Control of Tropical Diseases, most available drugs are associated with prolonged treatments, high toxicity, and the emergence of drug resistance or a lack of treatment adherence and, therefore, there is a need for new drugs [8]. In recent years, non-profit research and development organizations, academic and institutional centers, and public–private partnerships with pharmaceutical companies have succeeded in discovering new drugs, which must be evaluated in various clinical phases [9].

The ongoing search for new drugs to treat different diseases has been focused on nature because natural sources have continuously provided humanity with broad and structurally diverse pharmacologically active compounds. These continue to be utilized as highly effective drugs to combat many deadly diseases, or as lead structures to develop novel-synthetically derived compounds. Traditionally, higher plants and, since the discovery of penicillins, terrestrial microorganisms have proven to be the richest sources of natural drugs that are indispensable for treating several diseases [10]. Natural products are a source of antiprotozoal drugs. For example, for malaria treatment, quinine was isolated from *Cinchona* species, and, later, artemisinin was obtained from *Artemisia annua* [11]. Although infrequently mentioned, the leishmanicidal agents, amphotericin B and paromomycin, are produced by the actinobacteria *Streptomyces nodosus* and *S. krestomuceticus*, respectively [12,13]. For this reason, the present review focuses on gathering information about metabolites produced by bacteria and fungi, which could be consideredas hits in the development and design of new drugs for the treatment of trypanosomatid diseases in the future.

## 2. Trypanosomatid Diseases

Leishmaniasis comprises a group of diseases with different clinical manifestations caused by various species of *Leishmania* parasites. Twenty species are known to cause infections and drive the four clinical forms of the disease: cutaneous, diffuse cutaneous, mucocutaneous and visceral leishmaniasis. Cutaneous forms are produced by *L*. *mexicana* or *L*. *braziliensis* complexes in the Americas and *L*. *major*, *L*. *tropica*, or *L*. *aethiopica* in the Old World. Cutaneous lesions resolve spontaneously after some months but, depending on the *Leishmania* species causing them, they can evolve into diffuse cutaneous and mucocutaneous leishmaniasis [14]. Visceral leishmaniasis is caused by *L. infantum* in Latin America [15], *L*. *donovani* in Africa and Asia, or *L*. *infantum* in the Mediterranean basin, which can be fatal if not treated [14].

Current chemotherapy for leishmaniasis has many drawbacks, including low efficacy, severe toxic side effects, and the appearance of drug resistance. The first effective drug, ureastibamine, was developed in India in 1922, but it had severe side effects. Later, the refinement and development of pentavalent antimonials reduced the side effects.These compounds remain crucial in the treatment of all forms of leishmaniasis. However, reports of non-response to pentavalent antimony began in the 1970s, even at higher doses, and several other side effects of this regimen were reported, including pancreatic inflammation, nausea, and abdominal pain, pancytopenia, peripheral neuropathy, and cardiotoxicity. This led to trials with pentamidine and amphotericin B. Pentamidine’s reported side effects were myalgia, pain at the injection site, nausea, headache, and, less commonly, an oral metallic taste, a burning sensation, numbness, and hypotension [16,17]. Amphotericin B is highly nephrotoxic and, to minimize these side effects, several formulations of colloids and lipids were prepared. These preparations are comparatively safe but extremely expensive. Later, miltefosine was introduced to the market, but the drug is teratogenic and, thus, the administration is contraindicated during pregnancy and shows severe gastrointestinal side effects. Moreover, its cost is another limiting factor. Finally, other drugs, such as paromomycin, allopurinol, and sitamaquine, have been reported with variable cure rates [17].

American trypanosomiasis (Chagas disease), caused by the protozoan parasite *Trypanosoma cruzi*, is characterized by a generalized infection that clinically courses from an acute form to a chronic phase. The chronic phase of the disease is highly disabling due to cardiac and digestive disorders that can eventually lead to death. This flagellated protozoan parasite is transmitted to humans by a blood-sucking reduviid bug, which deposits its infective feces on the skin at the time of biting. It can also be transmitted directly by infected blood or by congenital transmission [6,18]. The drugs currently used for Chagas disease’s etiological treatment are nitroimidazole, benznidazole, and nitrofuran, nifurtimox. The benefits of benznidazole are most significant during the acute stages of the disease in adults and children, and young adults with intermediate Chagas disease [19]. Benznidazole prevents congenital transmission when administered to reproductive-age women, which may be an essential strategy to prevent disease in newborns [20]. Tolerance to benznidazole is satisfactory since no severe side effects have been observed in treated patients. Side effects include allergy, skin disease, nausea, and vomiting. Less common are polyneuropathy and bone marrow depression. Nifurtimox is used as a second-line option for the treatment of this disease. Several clinical studies have shown that this drug, in children and adults, achieved a cure rate of 80–90%. However, its adverse side effects are common and include anorexia, vomiting, gastric pain, insomnia, headache, myalgia, and seizures [21,22].

Following the bite of the tsetse fly of the genus *Glossina*, Human African trypanosomiasis (HAT) can occur in two clinical forms: a chronic form caused by *Trypanosoma brucei gambiense*, found mainly in West and Central Africa, representing more than 98% of recorded cases, and an acute form, caused by *Trypanosoma brucei rhodesiense*, found mainly in Eastern and South-Central Africa. Without treatment, both types of parasites penetrate the blood–brain barrier and invade the CNS, manifesting in complex symptoms that lead to patient death [23]. Drug treatment in the early stage of HAT is effective and less toxic than in the late stage. Pharmacotherapy for *T*. *b*. *gambiense* consists of intramuscular or intravenous pentamidine. For late-stage disease, the first-line therapy for *T*. *b*. *gambiense* is NECT, a combination of intravenous eflornithine (DFMO), an ornithine decarboxylase inhibitor, and oral nifurtimox [24]. Finally, fexinidazole, a derivative of 5-nitroimidazole, is a DNA synthesis inhibitor developed by Sanofi in collaboration with the Drugs for Neglected Diseases initiative (DNDi) for the treatment of HAT [25]. Fexinidazole is the first oral drug treatment for the disease’s early and late stages [26,27].

## 3. Population Affected by these Diseases

The World Health Organization (WHO) has reported that leishmaniasis affects 98 countries, with between 12 and 15 million people infected. Additionally, around 50,000 to 90,000 new cases of visceral leishmaniasis and 600,000 to 1 million cases of cutaneous leishmaniasis are reported annually worldwide, leading to between 26,000 and 65,000 deaths [28,29]. There is evidence that *Leishmania*-HIV co-infections are becoming a significant health problem in affected areas [30].

Chagas disease is an endemic disease widespread in 21 countries from the southern United States to southern Argentina. It has been estimated that around 6–7 million people are infected with *T. cruzi*, the agent responsible for this disease, with an annual average incidence of 30,000 new cases and 14,000 deaths. About 70 million people live in exposure areas and are at risk of contracting this disease [31]. Finally, HAT is a daily threat to more than 60 million people in 36 Sub-Saharan African countries, including 22 of the world’s least-developed countries. Sustained control efforts have reduced the number of new cases. In 2009, the number of reported cases fell below 10,000 for the first time, and only 977 cases were recorded in 2018 [32].

## 4. Microbial Diversity as a Source of Antiprotozoal Metabolites

Natural resources are recognized as important sources of potential drugs for treating various infections, and microorganisms are a rich natural source of diverse compounds [33]. The discovery of penicillin from *Penicillium notatum* marked a significant shift from plants to microorganisms as a source of natural products. The early years of antibiotic research discovered streptomycin from *Streptomyces griseus*, cephalosporin C from *Cephalosporium acremonium*, erythromycin from *Saccharopolyspora erythraea*, and vancomycin from *Amycolatopsis orientalis* [34]. Moreover, it is worth noting that two drugs used to treat leishmaniasis, amphotericin B and paromomycin, were isolated from *S*. *nodosus* and *S*. *krestomuceticus*, respectively [12,13].

To the best of our knowledge, few reviews are deal with bacterial and fungal metabolites against trypanosomatid parasites [11,35,36,37] since these reviews only briefly mention them.

## 5. Bacterial Metabolites

In nature, most bacteria exist attached to surfaces within biofilms and are inherently different from those in the planktonic state, due to a change in their metabolism. Thus, these microorganisms produce anti-predator secondary metabolites, which may be considered as potential new bioactive products [38].

### 5.1. Actinobacteria

**Figure 1:**
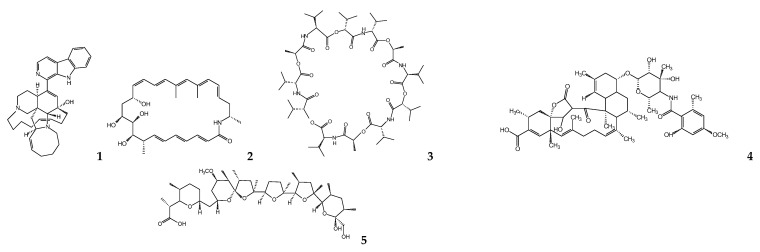

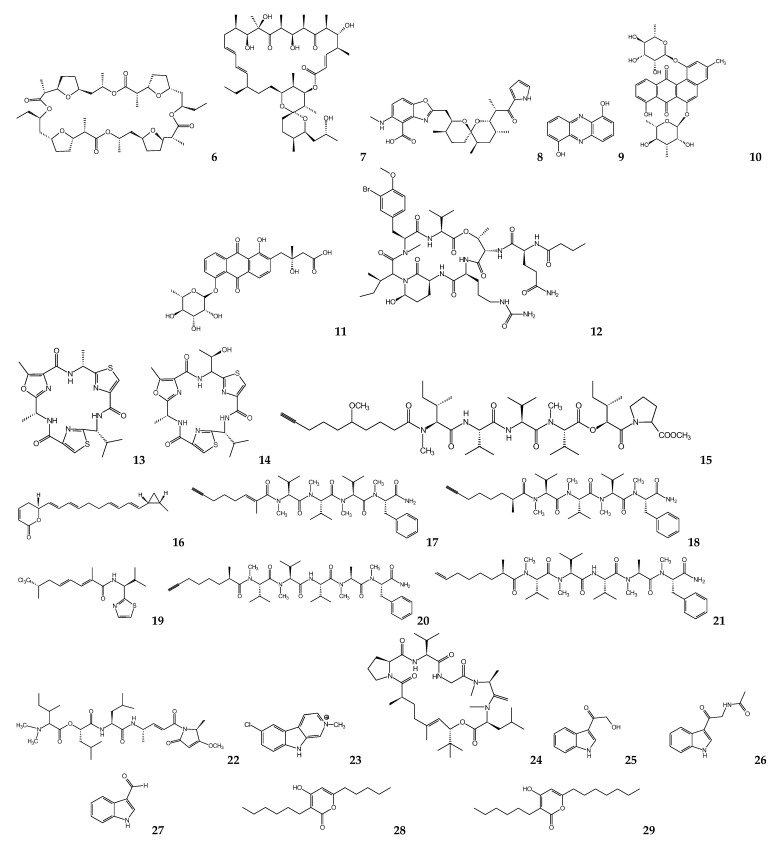
Chemical structures of bacterial metabolites with antitrypanosomatid activity.

Among these microorganisms, actinomycetes abundant in soil and marine organisms are well-known producers of a wide range of bioactive secondary metabolites and antibiotics [33]. Manzamines are complex polycyclic marine-derived alkaloids that possess a fused and bridged tetra- or pentacyclic ring system attached to a β-carboline moiety [39]. Manzamines have been found in several species belonging to the Chalinidae, Niphatidae, Petrosidae, Theorectidae, and Irciniidae families of marine sponges worldwide [39,40]. To date, approximately 40 related compounds have been reported, with manzamine A (**1**) (Figure 1) being the most important. This metabolite exhibited a 50% inhibitory concentration (IC_50_) of 1.63 μM against the promastigotes of *L*. *donovani* as well as an IC_50_ of 2.18 μM against mammalian kidney fibroblasts (Vero cells), demonstrating only slight selective activity against the parasites [41]. Studies on the structure–activity relationship established that, for leishmanicidal activity, the C-12 hydroxy, C-34 methine, or lower aliphatic ring conformation are indispensable, whereas the β-carboline moiety is not essential [41,42]. Finally, because of the presence of manzamines in multiple sponge species with wide geographical distribution, it has been suggested that manzamine could be of microbial origin. This was demonstrated when an actinomycete *Micromonospora* sp., which produces manzamine A and 8-hydroxy-manzamine, was isolated from the sponge *Acanthostrongylophora ingens* [40,43]. Additionally, from *Micromonospora* sp., lobosamide A (**2**), which is a 26-membered macrolactam, was isolated and exhibited activity against *T*. *b*. *brucei* at 0.8 μM and low cytotoxicity to T98G cells (>66 μM). The structure–activity relationship with lobosamide A analogs established that the isomerization of the double bond between carbons C-14 and C-15 is unfavorable. In contrast, a hydroxyl group at C-10 and the methylation pattern at carbons C-8 and C-20 are significant for trypanocidal activity [44].

Valinomycin (**3**), a cyclodepsipeptide recovered from various soil-derived actinomycetes (*Streptomyces fulvissimus*, *S*. *roseochromogenes*, and *S*. *griseus* var. *flexipertum*) was isolated from a marine *Streptomyces* sp. found in the sponges *Axinella polypoides* and *Aplysina aerophoba*. This metabolite exhibited significant inhibitory activities against the parasites *L*.*major* (IC_50_ < 0.11 μM) and *T*. *b*. *brucei* (IC_50_ = 0.0032 μM). Additionally, it exhibited cytotoxicity against 293T kidney epithelial cells (IC_50_ = 11.2 μM) and J774.1 macrophages (IC_50_ < 0.1 μM). Its biological activity could be due to the nonpolar portion of the molecule, which behaves similar to an ionophore that modulates the transport of ions such as potassium, across biological membranes [45].

The actinobacterium *S. axinellae* isolated from the sponge *Axinella polypoides* produced five compounds, called tetromycins. These compounds were active against the trypomastigotes of *T*. *b. brucei* but were also toxic to 293T kidney cells and J774.1 macrophages, excluding tetromycin 1 (**4**), which had an IC_50_ of 31.7 µM and no cytotoxic activity to both cell lines at a concentration of 100 µM [46].

Several actinobacteria strains isolated from ant exoskeletons have recently emerged as a prolific and underexplored source of microbial compounds. The bacterium, *Streptomyces* sp. ICBG292, isolated from *Cyphomyrmex* exoskeletons, produced a polyether antibiotic known as nigericin (**5**), which was first isolated from *S*. *hygroscopicus* in the 1950s [47]. This metabolite was active against *L. donovani* promastigotes and amastigotes, with IC_50_ values of 0.28 μM and 0.13 µM, respectively. Additionally, *Streptomyces* sp. ICBG233, isolated from *Atta sexdens* exoskeletons, generated a macrotetrolide, called dinactin (**6**), which also exhibited activity against *L. donovani* promastigotes and amastigotes at IC_50_ values of 0.03 μM and 0.02 μM, respectively. These metabolites were more active than the positive control (miltefosine) and, although they presented cytotoxic activity against THP-1 cells, they had high selectivity indexes, 89 for nigericin and 656 for dinactin [48]. Finally, these compounds are considered ionophores that bind reversibly and transport ions across biological membranes. Nigericin moves sodium and potassium ions across membranes [49]. Dinactin is a member of the nactin family, with the ability to selectively complex a wide variety of cations [48].

Oligomycin (**7**), a 26-membered macrolactone with a spiroketal ring isolated from *Streptomyces diastatochromogenes*, exhibited activity against *T*. *brucei* S42 at 3.8 μM, causing the inhibition of glucose utilization, since pyruvate production was not observed, as well as oxygen consumption [50,51]. Later, it was determined that oligomycin inhibits mitochondrial ATP synthase by promoting the induction of glucose uptake through the AMP-activated protein kinase (AMPK) and protein kinase B (Akt) pathways while preventing the intracellular uptake of calcium ions [52]. Finally, this metabolite exhibited activity against amastigotes forms of the *T*. *cruzi*, expressing galactosidase (Tulahen strain) in Vero cells at an IC_50_ of 0.52 μM. However, in vivo studies with Balb/c mice were not performed due to its toxicity [53].

Calcimycin (**8**) (antibiotic A23187), a carboxylic polyether isolated from *Streptomyces chartreusensis* [54] and a selective ionophore for divalent cations, particularly Ca^2+^, Mg^2+^, and Mn^2+^ [55], demonstrated activity against intracellular amastigotes of *Leishmania enriettii* at concentrations between 0.01–0.25 μM, by observing the decreased incorporation of ^3^[H]-thymidine into parasites released from sodium dodecyl sulfate (SDS)-lysed and lipopolysaccharide(LPS)-stimulated macrophages [56]. Subsequently, in another study, the pretreatment of peritoneal macrophages from Balb/c mice with calcimycin at a concentration of 1 μM for 48 h before their infection with *Leishmania major* led to a decrease in the incorporation of ^3^[H]-thymidine by intracellular amastigotes [57]. Its action mechanism was recently confirmed in *L*. *major* promastigotes co-incubated with calcimycin by evaluating the activity with resazurin. Calcimycin showed a dose-dependent effect with an IC_50_= 0.16 μM. In this study, it was shown that, at lower concentrations, calcimycin had a cytostatic effect and, at higher concentrations, a cytotoxic effect, causing the cell death of *Leishmania* parasites due to the loss of mitochondrial polarization and plasma membrane integrity, which can be blocked by specific inhibitors of constitutive Ca^2+^/calmodulin nitric oxide synthase [58].

The co-culture of two sponge-derived actinomycetes, *Actinokineospora* sp. EG49 and *Nocardiopsis* sp. RV163 produced 1,6-dihydroxyphenazine (**9**), which was active against *T*. *brucei* parasites with an IC_50_ value of 19 μM [59]. Additionally, the *O*-glycosylated angucycline, actinosporin A (**10**), isolated from the culture of *Actinokineospora* sp. EG49 exhibited activity against *T*. *b*. *brucei* with an IC₅₀ of 15 µM [60].

From solid cultures elicited with the *N*-acetyl glucosamine of *Actinokineospora spheciospongiae*, isolated from the sponge *Spheciospongia vagabunda*, produced an anthraquinone called fridamycin H (**11**), which showed activity against *Trypanosoma brucei* strain TC 221 after 48 and 72 h with IC_50_ values of 7.18 and 3.35 μM, respectively. Moreover, it did not present cytotoxic activity against J774.1 macrophages with an IC_50_ of > 200 μM. Therefore, this strategy of inducing compounds by elicitation may allow for new bioactive chemical scaffolds [61].

### 5.2. Cyanobacteria

Marine cyanobacteria are a source of many novel natural product structures, some of which possess highly potent biological properties. Several metabolites are partially composed of amino acids, and these are often integrated with sections of polyketides to produce various nitrogen-rich lipids. Oxidations, methylations, and even halogenations often modify the chemical structures of these compounds. Symplocamide A (**12**), a bromide depsipeptide isolated from a marine cyanobacterium, *Symploca* sp., showed antiprotozoal activity against *L*. *donovani* and *T*. *cruzi* (IC_50_ > 9.5 µM). However, it is also a highly cytotoxic metabolite against NCI-H460 (non-small cell lung cancer) and neuro-2A (mouse neuroblastoma) cell lines [62].

The cyclic hexapeptides, venturamides A and B, isolated from the marine cyanobacterium *Oscillatoria* sp. showed weak selectivity for the *L. donovani* and *T. cruzi* parasites versus mammalian host cells. Venturamide A (**13**) showed in vitro activity against *T. cruzi* (IC_50_ = 14.6 μM), with only mild cytotoxicity to mammalian Vero cells (IC_50_ = 86 μM). Venturamide B (**14**) displayed antiprotozoal activity against *T. cruzi* at an IC_50_ of 15.8 μM, and mild cytotoxicity to Vero cells (IC_50_ = 56 μM) [63].

Another species of *Oscillatoria*, *O. nigro-viridis*, isolated as an epiphytic species from a partially purified culture of cyanobacterium *Lyngbya majuscula*, was active against axenic amastigotes of *L. mexicana* and amastigotes of *T. cruzi* in Vero cells. Its lipodepsipeptide, viridamine A (**15**), had antileishmanial activity at an IC_50_ of 1.5 μM and antitrypanosomal activity at an IC_50_ of 1.1 μM [64].

Coibacin A (**16**), a polyketide α,β-unsaturated-δ-lactone with a methyl cyclopropyl ring in its linear chain, isolated from the marine cyanobacterium *Oscillatoria* sp., exhibited selective activity to axenic amastigotes of *L. donovani* at a concentration of 2.4 μM and showed cytotoxic activity against NCI-H460 human lung cancer cells at 31.5 μM. However, coibacin A did not show activity against intracellular amastigotes of *Leishmania mexicana* in RAW264.7 mouse macrophage cells, possibly due to not being able to cross the plasma membrane of these cells [65].

A linear peptide, dragonamide E (**17**), and two known linear peptides, dragonamide A (**18**) and herbamide B (**19**), were isolated from an extract of marine cyanobacterium*Lyngbya majuscula*. These compounds were active against the *L*. *donovani* axenic amastigote with an IC_50_ between 5.1 and 6.5 μM, and this activity is due to the presence of a residue containing an aromatic ring at the end of the peptide [66]. Other metabolites of *L*. *majuscula* closely related to dragonamide A, almiramides B (**20**) and C (**21**), which contain an extra Ala residue, no methyl group on Val 1, and the opposite configuration of the α-carbon of the lipophilic side chain, showed better antileishmanial activity against *L*. *donovani* with IC_50_ values of 2.4 and 2 μM, respectively. However, these compounds were also cytotoxic to Vero cells [67,68]. Despite significant structural similarities between these compounds, an unsaturated terminus on the lipophilic side chain seems to play a critical role in antileishmanial activity in dragonamides and almiramides [67]. Improved leishmanicidal activity is also due to the methylation patterns that determine the degree of membrane permeability [68].

Another linear peptide, gallinamide A (**22**), isolated from the organic extract of a marine cyanobacterium, *Schizothrix* sp., was moderately active against *L*. *donovani* (IC_50_ = 9.3 μM) and showed in vitro cytotoxicity toward Vero cells (TC_50_ = 10.4 μM). However, it was not toxic against NCI-H460 human lung tumor and neuro-2a mouse neuroblastoma cell lines at 17 μM. This metabolite contains the unusual 4-(*S*)-amino-2-(*E*)-pentenoic acid subunit and the presence of a methyl-methoxypyrrolinonemoiety at the C-terminus, and its leishmanicidal activity is related to the presence of a terminal *N*,*N*-dimethyl-isoleucine group [69].

The β-carboline alkaloid, nostocarboline (**23**), derived from the cyanobacterium *Nostoc* 78-12A, was synthetized and evaluated against different parasites. Against axenic amastigotes of *L. donovani*, the alkaloid presented an IC50 value of 34.3 μM, whereas its cytotoxicity in the L6 myoblast cells of rats was 121 μM [70].

A cyclic depsipeptide, named janadolide (**24**), was isolated from a marine cyanobacterium *Okenia* sp. This metabolite exhibited potent antitrypanosomal activity with an IC_50_ of 47 nM against the *T*. *brucei* GUTat 3.1 strain and had no cytotoxic effect against MRC-5 cells (>10 μM) [71]. In attempting to synthesize this compound, which is a 23-membered macrocyclic and a rare polyketide–peptide hybrid containing a tert-butyl group, it was observed that this functional group is essential for parasitic activity [72]. Recently, other researchers synthesized janadolide, which had in vitro antitrypanosomal activity against the pathogenic parasites *T*. *b. rhodesiense* (STIB900 strain) and *T*. *cruzi* (Tulahuen C4 strain) with IC_50_ values of 91.3 and 69.3 μM, respectively. Additionally, eight analogs were synthesized, which were not cytotoxic to human L6 cell lines at high concentrations between 100–150 μM. The structure–activity relationship suggests that the replacement of the olefin moiety and ester bond with amide bonds does not compromise the compound’s activity [73].

### 5.3. Firmicutes

The bacterium *Bacillus pumilus* isolated from the coral mucus *Antiphates* sp. produced three indole alkaloids with selective activity against *T. cruzi* amastigotes expressing β-galactosidase (Tul-β-Gal) in Vero cells. The compounds 3-hydroxyacetylindole (**25**), *N*-acetyl-β-oxotryptamine (**26**), and 3-formylindole (**27**) presented similar trypanocidal activities with IC_50_ values of 20.6, 19.4, and 27 μM, respectively. Likewise, the compounds *N*-acetyl-β-oxotryptamine and 3-formylindole showed no significant cytotoxic activity against Vero cells. Due to their similarity in their chemical structures, it is possible that the presence of a carbonyl group in C-1 of the lateral chain, being an electron-attracting group, is responsible for the antiprotozoal activity [74].

### 5.4. Gammaproteobacteria

Polyketides, such as the 3,6-dialkyl-4-hydroxy-2-pyrone marine metabolites, pseudopyronines A (**28**) and B (**29**), isolated from the fermentation broth of *Pseudomonas* sp., were synthesized, and evaluated against *L. donovani* axenic amastigotes, *T. b. rhodesiense* trypomastigotes, and *T. cruzi* amastigotes. Both metabolites had leishmanicidal activity with IC_50_ values of 9.8 and 4.65 μM, respectively, and weak cytotoxic activity against the L6 rat skeletal myoblast cell line; their selectivity indices were 8.8 (pseudopyronine A) and 13 (pseudopyronine B). However, these metabolites had no significant activity against *Trypanosoma* species. Additionally, a small set of structurally related compounds were evaluated against these parasites, which were more active against *L. donovani*. A probable action mechanism of these metabolites is the inhibition of enzymes involved in fatty acid biosynthesis in the protozoan parasites [75].

## 6. Fungal Metabolites

### Ascomycetes

**Figure 2:**
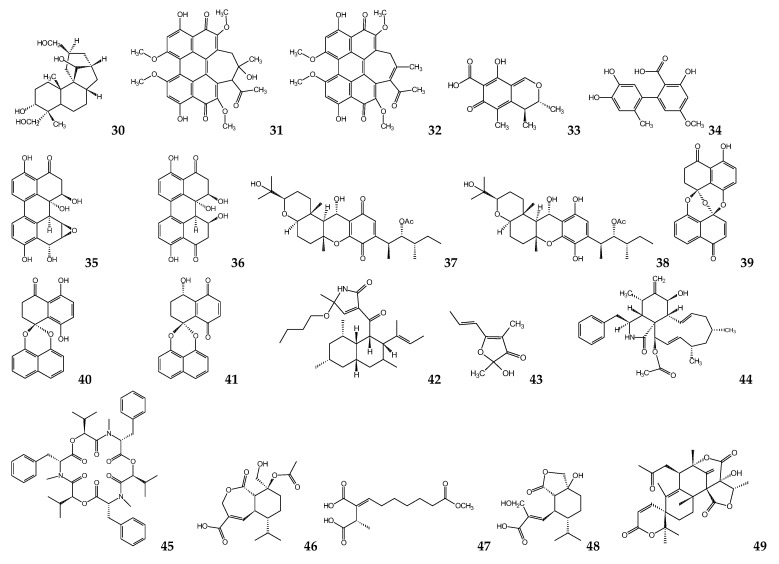

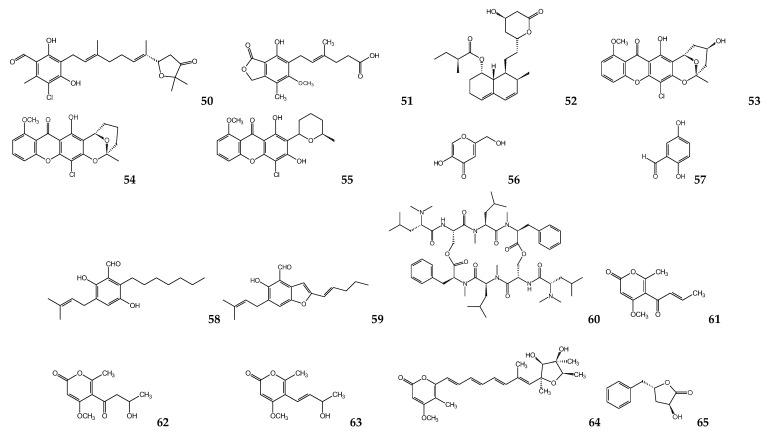
Chemical structures of fungal metabolites with antitrypanosomatid activity.

Fungal secondary metabolites possess broad bioactive applications, and different classes of fungal metabolites have antiprotozoal potential. Aphidicolin (**30**) (Figure 2), a tetracyclic diterpene antibiotic isolated from the fungus *Nigrospora sphaerica*, was tested against *Trypanosoma* spp. and *Leishmania* spp. At 0.3 µM, it inhibited cell division but did not inhibit the DNA synthesis of *T*. *brucei* bloodstream forms. Higher concentrations of aphidicolin (3 and 29 μM) were needed to inhibit DNA synthesis, and the cells failed to resume growth after removing the drug [76]. Concerning *Leishmania* parasites, aphidicolin has been evaluated against the promastigote forms of *Leishmania donovani*, *L*. *infantum*, *L*. *enrietti*, and *L*. *major*, which were active in all of them with mean effective doses (EC_50_) that ranged between 0.41 and 1.6 μM. Additionally, it did not present toxic activity against two neoplastic cell lines (squamous carcinoma (KB) and melanoma (SK-Mel)) and murine bone marrow-derived macrophages as host cells (BMMØ). Remarkably, against *L*. *donovani* amastigotes in BMMØ cells, it had an EC_50_ of 0.35 μM, with a selectivity index towards parasites. Although aphidicolin possesses the selective inhibition of leishmanial and mammalian DNA polymerases, the specific differences in the target enzyme DNA polymerase between mammalian cells and trypanosomatid parasites give a plausible explanation for the different activities of these compounds against host cells and *Leishmania* parasites [77].

Hypocrellin A (**31**) and hypocrellin B (**32**), perylene quinonoid pigments isolated from fungus *Hypocrella bambusae*, exhibited antileishmanial activity. Hypocrellin A showed significant antileishmanial activity against promastigotes of *L*. *donovani* (IC_50_= 0.5 μM), while B was moderately active with an IC_50_ of 24 μM. Interestingly, the antileishmanial activity of hypocrellin A was three- and sixfold more potent than that of amphotericin B and pentamidine, respectively [78].

Endophytes are microorganisms, including bacteria or fungi, which live within apparently healthy host plant tissues without causing observable manifestations of the disease. Endophytic microbes have been recognized as essential sources of structurally new and biologically active secondary metabolites [79]. The polyketide citrinin (**33**), produced for an endophytic fungus *Penicillium janthinellum,* isolated from fruits of *Melia azedarach* (Meliaceae), presented 100% activity against promastigotes of *L. mexicana* after 48 h at a concentration of 160 µM [80].

Among several endophytic fungi isolated from *Trixis vauthieri* (Asteraceae) leaves, *Alternaria* sp. produced a biphenyl phenolic compound, called altenusin (**34**), that demonstrated inhibitory activity against the enzyme trypanothione reductase (TR) from *T*. *cruzi* at an IC_50_ of 4.3 μM. This enzyme is a validated drug target in trypanosomatids as it was shown to be essential for the survival of these parasites, protecting them against oxidative stress [81]. However, this inhibitory activity did not translate into the decreased viability of amastigote-like forms of *L*. *amazonensis*, probably because altenusin did not reach the intracellular compartments where TR is located [82].

In another study, two new perylenequinones were isolated from *Alternaria* sp. (DC401), an endophytic fungus isolated from *Pinus ponderosa* (Pinaceae). These compounds were tested for their in vitro antileishmanial potential, where 3,6,6a,9,10-pentahydroxy-7,8-epoxy-4-oxo-4,5,6,6a,6b,7,8,9-octahydroperylene (**35**) and 3,6,6a,7,10-pentahydroxy-4,9-dioxo-4,5,6,6a,6b,7,8,9-octahydroperylene (**36**) showed antileishmanial activity against *L*. *donovani* promastigotes with IC_50_ values of 7 and 12 μM, respectively. However, **36** exhibited cytotoxicity activity against Vero cells with an IC_50_ of 10 μM [83].

The endophytic fungus, *Cochliobolus* sp., isolated from *Piptadenia adiantoides* (Fabaceae), produced two quinonoid compounds, cochlioquinone A (**37**) and isocochlioquinone A (**38**). Both compounds showed antileishmanial activity against axenic amastigotes of *L*. *amazonensis*, with EC_50_ values of 1.7 and 4.1 μM, respectively. These compounds were not active against three human cancer cell lines (MCF-7, TK-10, and UACC-62), indicating some degree of selectivity towards the parasites. However, these metabolites were not active against trypanothione reductase (TR) from *T. cruzi* [84].

From *Edenia* sp., a fungus isolated from a mature leaf of *Petrea volubilis* (Verbenaceae), bisnaphthospiroketal-containing antileishmanial compounds were purified. Preussomerin EG1 (**39**) (IC_50_ = 0.12 μM) was the most active and inhibited growth of *L. donovani* axenic amastigotes with a similar potency to amphotericin B (IC_50_ = 0.1 μM). This compound showed marked cytotoxicity to mammalian Vero cells (IC_50_ = 9 μM), although it was 75-fold more active against *L. donovani*. Palmarumycin CP_17_ (**40**) (1.34 μM) and palmarumycin CP_18_ (**41**) (0.62 μM) were less active than preussomerin EG1, but they also showed less cytotoxicity to mammalian Vero cells, thus giving therapeutic indexes of 130 and 245, respectively [85]. Furthermore, palmarumycin CP_18_ showed antileishmanial activity against *L*. *donovani* amastigotes in periperitoneal macrophage (Balb/c mice), with IC_50_ values of 23.5 µM [86].

The marine endophytic fungus *Ascochyta salicorniae*, isolated from the green alga *Ulva* sp., produced a pyrrole alkaloid, ascosalipyrrolidinone A (**42**). This metabolite exhibited significant activity against *T*. *cruzi* at a minimum inhibitory concentration (MIC) of 2.6 μM and against *T*. *b*. *rhodesiense* at a MIC of 70.1 μM, as well as having cytotoxic activity against rat skeletal muscle myoblast cells (MIC = 8.65 μM) and mouse peritoneal macrophages (MIC = 5.1 μM). Additionally, the furanic metabolite isolated from *A. salicorniae*, 2,3-dihydro-2-hydroxy-2,4-dimethyl-5-*trans*-propenylfuran-3-one (**43**), exhibited activity against *T*. *b*. *rhodesiense* and *T*. *cruzi* at MIC values of 178 and 535 μM, but it was more cytotoxic to L-6 cells (MIC = 59.4 μM) [87].

Compound 18-des-hydroxy cytochalasin H (**44**) produced by the endophytic fungus *Diaporthe phaseolorum*-92C, isolated from the roots of *Combretum lanceolatum* (Combretaceae), reduced the viability of *L*. *amazonensis* promastigotes, IC_50_ = 19.2 μM, and of the cancer cells MDA-MB-231 and MCF-7 with IC_50_ values of 36.5 and 18.3 μM, respectively, and gave an IC_50_ of 428 μM for the cytotoxicity towards normal cells GM07492A [88].

A screening of 82 endophytic fungi from stems and barks of *Caesalpinia echinata* Lam. (Fabaceae) resulted in the isolation of *Fusarium* sp. KF611679, which produced a depsipeptide called beauvericin (**45**) with activity against *T*. *cruzi* amastigotes with an IC_50_ of 2.43 μM, and an IC_50_ of 6.38 μM against the mouse L929 fibroblasts [89]. The endophytic fungus *Nectria pseudotrichia*, also isolated from the tree *C. echinata*, biosynthesized active metabolites against intracellular amastigote forms of *L. braziliensis* expressing firefly luciferase. Compounds, 10-acetyl trichoderonic acid A (**46**), 6′-acetoxy-piliformic acid (**47**), and hydroheptelidic acid (**48**) were more active, with IC_50_ values of 21.4, 28.3, and 24.8 µM, respectively, and showed low toxicity to Vero and THP-1 cells [90].

Two endophytic strains isolated from *Handroanthus impetiginosus* (Bignoniaceae) leaves, identified as *Talaromyces purpurogenus* H4 and *Phanerochaete* sp. H2 (Basidiomycete) were grown in mixed and axenic cultures. The meroterpenoid austin (**49**) was detected only in the extracts from the mixed culture. This metabolite had an IC_50_ of 73.1 μM against epimastigotes of *T*. *cruzi* and a 50% cytotoxic concentration (CC_50_) of 351 μM on H9c2 cells, so it had a selectivity index of 4.8 [91].

Ascofuranone (**50**), a meroterpenoid produced by several filamentous fungi, including *Acremonium egyptiacum* (synonym: *Acremonium sclerotigenum*), which has been known as *Ascochyta vicia* [92,93], specifically inhibited the ubiquinol oxidase activity of trypanosoma mitochondrial (AOX or TAO) at an IC_50_ of 0.13 nM [93,94]. Additionally, ascofuranone showed therapeutic efficacy against *T*. *b*. *brucei* infection in mice at 100 mg/kg/day intraperitoneally for four consecutive days and 400 mg/kg/day for eight days orally. It was also determined that TAO activity decreased by 30% [95]. Since *T*. *brucei* parasites rely exclusively on glycolysis as an energy source in the mammalian bloodstream using TAO to reoxidize NADH and mammalian hosts lack this protein, this enzyme is considered a key target for the generation of antitrypanosomal drugs [96]. The structure–activity relationship analysis of ascofuranone demonstrated that 1-formyl and 6-hydroxyl groups are responsible for the interaction with the enzyme, and 2-methyl and/or 3-chloro groups contribute to proper conformation, while the furanone ring is not essential for activity [94]. However, its stereoselective synthesis is complex, and, therefore, a bioengineering strategy has recently been proposed to benefit combinatorial biosynthesis through biocatalysts [92,97].

Mycophenolic acid (**51**) produced by several *Penicillium* species showed inhibitory activity against the inosine 5’-monophosphate dehydrogenase (IMPDH) of *T*. *brucei* at a Ki value of 21 nM [98]. Mycophenolic acid also demonstrated an inhibition rate against *T*. *congolense* (99.60%), *T*. *b*. *brucei* (82.99%), and *T*. *evansi* (90.53%) at a concentration of 1 μM, showing that it inhibited the IMPDH of *T*. *congolense* [99]. This enzyme is crucial in *Trypanosoma* spp. because it lacks a de novo purine synthesis pathway, making purine nucleotide synthesis in these parasites solely dependent on a salvage pathway [99]. Finally, a recent study showed activity against *T*. *b*. *brucei* at an IC_50_ of 0.51 μM and *T*. *cruzi* intracellular amastigotes at an IC_50_ of 1.6 μM, but it exhibited cytotoxic activity against macrophages [100].

Mevastatin (**52**), the first statin isolated from *Penicillum citrinum* [101], was active against promastigotes and intracellular amastigotes of *Leishmania donovani* with IC_50_ values of 23.8 and 7.5 µM, respectively, without showing toxicity to the THP-1 macrophages. Mevastatin-treated parasites showed a 66% reduction in ergosterol levels and it also induced morphological changes in the parasites accompanied by lipid body accumulation. Therefore, its antileishmanial effect was due to the inhibition of 3-hydroxy-3-methyl glutaryl-CoA reductase (HMGR), which eventually leads to a reduction in ergosterol levels and parasites death [102].

Xanthones represent a structurally diverse group of natural products with a broad range of biological activities. Several derived xanthones were isolated from higher plants, lichens, and fungi and possess promising antiprotozoal activities, but they also showed pronounced cytotoxicity, making them problematic for pharmaceutical use. Investigations of the marine-derived fungus *Chaetomium* sp. yielded three new xanthones, called chaetoxanthones A (**53**), B (**54**), and C (**55**), with unusual and rare structural features for this structural class of natural products. Compound A was active against trypomastigotes of *T*. *b*. *rhodesiense* at an IC_50_ of 12.6 μM, whereas compound C inhibited the growth of *T*. *cruzi* amastigotes with an IC_50_ value of 3.83 μM. Xanthones B and C showed leishmanicidal effects toward amastigotes of *L*. *donovani* with IC_50_ values of 9.6 and 8 μM, respectively, and only low cytotoxicity for compound C (IC_50_ = 119 μM) and no observed cytotoxic effects for compound B up to 254 μM [103].

Kojic acid (**56**) (KA) is a fungal metabolic product produced by a few species of *Aspergillus*, especially by *A*. *oryzae,* as a by-product in the fermentation process of malting rice [104], and it is widely used in cosmetics as a UV protector, hyperpigmentation suppressant and limiter of melanin formation [105]. In a study in vitro on promastigotes and amastigotes of *L*. *amazonensis*, KA exhibited an IC_50_ of 239 μM against promastigotes and an IC_50_ of 193 μM against amastigotes. Ultrastructural analysis of KA-treated amastigotes showed vesicle body presence in the flagellar pocket and an intense intracellular vacuolization and swelling of the mitochondrion. Additionally, it was observed that, after four weeks of treatment with KA (100 mg/kg/day) to infected Golden hamsters, collagen fiber production was increased, and the parasitic burden was drastically reduced [106]. In another study, it was demonstrated that KA promotes monocytes’ differentiation into macrophages and acts as an immunomodulator [107].

Filamentous fungus *Geosmithia langdonii* cultivated in potato dextrose broth, produced seven active compounds against *L*. *donovani* promastigotes, whose IC_50_ values ranged from 3.3 to 47.3 μM. In particular, 2,5-dihydroxybenzaldehyde (**57**) was the most active and had an antiprotozoal activity very similar to the positive control, pentamidine, which presented an IC_50_ of 3.2 μM [108]. Additionally, other metabolites produced by this fungus have been reported, such as carbasugar-type and diarylmethane compounds with activity against *L*. *donovani* [109,110].

The fungus *Eurotium repens* produced eight benzyl derivatives that were tested in vitro against *L*. *donovani* promastigotes. Six of them presented antiprotozoal activity with IC_50_ values ranging from 20.7 to 75.5 μM, with auroglaucin (**58**) and 2-(2′,3-epoxy-1′,3′-heptadienyl)-6-hydroxy-5-(3-methyl-2-butenyl)benzaldehyde (**59**) being the most active with IC_50_ values of 25 and 20.7 μM, respectively. These metabolites did not show cytotoxic activity to Vero cells [111].

IB-01212 (**60**), an antitumoral depsipeptide isolated from the mycelium of the marine fungus *Clonostachys* sp., has been shown to have leishmanicidal activity against *L*. *donovani* promastigote and *L*. *pifanoi* amastigote forms at LC_50_ values of 10.5 and 7.1 μM, respectively. This compound induces an apoptosis-like process without significant permeabilization of the plasma membrane, thereby suggesting an intracellular target associated with the depolarization of the mitochondrial electrochemical gradient. The evaluation of its synthetic analogs established that the cycle’s size, the preservation of the C-2 symmetry, and the nature of the bonds linking the two tetrapeptide halves participate in modulating the leishmanicidal activity [112].

Pyrenocines A (**61**), B (**62**), I (**63**), and citreoviridin (**64**) were isolated from the culture broth of *Paecilomyces* sp. FKI-3573. All compounds exhibited in vitro antitrypanosomal activity using GUTat 3.1 strain of *T. b*. *brucei* and slightly more selective activity towards parasites than MRC-5 cells. However, citreoviridin (IC_50_ = 1.2 μM) was the compound that showed the best selective index of 94. Since citreoviridin inhibited the mitochondrial F1-ATPase of *T. cruzi* and the structural similarity of this compound and pyrenocines, it was suggested that pyrenocines might perform their antitrypanosomal activity through a similar mode of action [113]. Finally, *Paecilomyces* sp. 7A22, a marine-derived fungus, excreteda polyketide called harzialactone A (**65**) in the culture medium. This metabolite showed activity against *L*. *amazonesis* promastigotes and intracellular amastigotes with IC_50_ values of 27.3 and 94.6 μM, respectively [114].

## 7. Future Microbial Metabolites

Microorganisms are promising resources for producing bioactive compounds, considering some of the advantages attributed to them, such as rapid and adaptable cultures and even the possibility of genetic manipulation. In the search for new biologically active molecules, research work has been extended to analyze microorganisms found in less explored environments. An example of this is the current trend of studying the microbiota of the vectors that transmit trypanosomatid diseases with the possible purposes of carrying out biological control or detecting antiprotozoal compounds [115]. *Enterobacter cloacae*, isolated from the digestive tract of *Lutzomyia evasi* (*Leishmania* vector), inhibited by 72.29% the growth of procyclic-like promastigotes when co-cultured under in vitro conditions. This study suggested that *E*. *clocae* generated peptides or molecules with cytolysin-like activity [115]. More recently, the methanolic extract of *Enterobacter hormaechei*, isolated from the intestine of *Lu*. *evasi*, showed toxic activity against promastigotes of *L*. *braziliensis* (UA301 strain) [116]. Studies into the vector microbiota of American and African Trypanosomiasis, triatomines and the tsetse fly, have been performed, but not yet with this objective; however, this may be a good strategy for finding new active compounds.

Marine ecosystems are complex and often harbor very diverse marine organisms. In this environment, there is intense competition for survival and environmental pressure, such as high salt content, high pressure, low temperature, oligotrophic characteristics, and lightless or high levels of solar radiation [9,117]. This biodiversity uses metabolites for defense, attack, or signaling [9]. These metabolites will continue to be a constant source of new chemical structures to be evaluated for potential therapeutic use, in their original form or after chemical optimization [118]. Currently, the study of culturable microbiota present in marine sediments at different depths and in ice fragments collected in Antarctica has shown that species of the genus *Penicillum* have preliminary activity against *L*. *amazonensis* promastigotes and *T*. *cruzi* trypomastigotes and amastigotes in L929 fibroblasts. Additionally, the ^1^H-NMR analysis determined the presence of aromatic compounds and terpenoids [119,120].

Another source of bioactive metabolites is endophytic microorganisms. Recent studies have shown that several fungal species produce metabolites with antiprotozoal activity, but very little has been reported about endophytic bacteria [121]. In this case, the isolation of endophytic bacteria from *Fagonia indica* (Zygophyllaceae) showed *Bacillus*, *Enterobacter*, *Pantoea*, *Erwinia,* and *Stenotrophomonas* species that exhibited preliminary activity against *Leishmania tropica* promastigotes [122].

However, drug discovery based on natural products presents a considerable challenge due to the large amount of resources and tedious purification protocols involved in identifying bioactive molecules from the highly complex mixtures that are the initial extracts. When purified, the isolated bioactive compound is often obtained in small quantities that are unlikely to span the entire drug discovery process [123]. Furthermore, there is controversy in evaluating these metabolites in a phenotypic or a target-based assay, although they are complementary, and both have their pros and cons [124].

In the phenotypic assay, it is necessary to use the most relevant form of the pathogenic parasite. In HAT, the bloodstream forms of *T*. *brucei* are accessible to culture under in vitro conditions that can closely resemble the blood environment where the parasite lives. However, this is not easy to reproduce with *T*. *cruzi*, as it has an early trypomastigote form in the bloodstream that rapidly invades different host cells and transforms into intracellular amastigotes. In contrast, in *Leishmania* spp., the most relevant pathological form is the amastigote that lives within the phagolysosomes of host macrophages, so compounds must cross multiple membranes to reach the parasite in cellular assays [9,124]. However, many compounds have been evaluated in forms of the free-living parasites, such as promastigotes and trypomastigotes, or axenic amastigotes, an unnatural extracellular form created under laboratory conditions, that can detect very preliminary antiprotozoal activity or simple cytotoxicity (Table 1) [9]. Therefore, it is necessary to culture mammalian cells infected in vitro with the pathogen, and, thus, the toxicity of the drug towards cells and its ability to be selective towards the parasites is known [125]. However, after a drug has demonstrated potential antiparasitic activity, its mechanism of action must be known, so target-based assays are indispensable.

In a target-based assay, it is established that if a validated drug target and an excellent chemical scaffold interact, a rational structure-based drug discovery approach can be adapted for the synthesis of thousands of new compounds in the search for drug candidates [9]. For neglected diseases in general, including trypanosomatid diseases, the success of target-based approaches has been minimal. In part, this reflects the absence of robustly validated targets, enzymes whose activity is essential for the parasite [124].

This is complicated by the presence of reactive compounds with multiple assay behavior, such as covalent bond formation, chelation, membrane perturbation, and redox activity, which are collectively known as Pan Assay Interfering Compounds (PAINS) [126]. Natural products that frequently contain PAINS include catechols, quinones, phenolic mannich bases, and hydroxyphenylhydrazones [127]. Despite a possible nano- or micromolar potency, PAINS lack a distinct biological mechanism, exhibit poor SAR or optimizing ability and, therefore, have minimal prospect for clinical development. Therefore, it is crucial to use assay techniques that eliminate PAINS and perform structural studies of hit binding to its target and structure–activity optimization studies of hits [128].

Finally, the search for new drugs against infectious diseases caused by trypanosomatids, considering the criteria proposed by the Drugs for Neglected Diseases initiative (DNDi) and the Global Health Innovative Technology (GHIT) Fund for hits compounds [129], should ideally have a potency that can be improved to generate a lead compound with the absence of highly reactive functional groups that could cause non-specific or false-positive results. Regarding its activity, it is recommended that a hit exhibits at least an IC_50_ value < 10 µM from phenotypic assays and a tenfold selectivity toward parasite cells over host organism cells. The cost of production is an essential variable. Therefore, the synthetic route should be as simple as possible (ideally five steps or less) with acceptable yields [129].

## Figures and Tables

**Table 1:** Microorganisms and their antiprotozoal metabolites.

Microbial Species	Metabolite(s)	Target Parasite	IC_50_ (μM)	Ref.
**Bacteria**				
**Actinobacteria**				
*Micromonospora* sp.	Manzamine A	*L. donovani* ^a^	1.63	[41,42]
*Micromonospora* sp.	Lobosamide A	*T. b. brucei* ^b^	0.80	[44]
*Streptomyces* sp.	Valinomycin	*L. major* ^a^ *T. b. brucei* ^b^	<0.11 0.0032	[45]
*Streptomyces axinellae*	Tetromycin 1	*T*. *b. brucei*^b^	31.7	[46]
*Streptomyces* sp. ICBG292	Nigericin	*L*. *donovani*^a,c^	0.28 ^a^ 0.13 ^c^	[48]
*Streptomyces* sp. ICBG233	Dinactin	*L*. *donovani*^a,c^	0.03 ^a^ 0.02 ^c^	[48]
*Streptomyces diastatochromogenes*	Oligomycin	*T*. *brucei*^b^ *T*. *cruzi*^c^	3.8 0.52	[50,53]
*Streptomyces chartreusensis*	Calcimycin	*L.enriettii*^c^ *L*. *major*^a^	0.01–0.25 0.16	[56,58]
*Actinokineospora* sp. EG49 and *Nocardiopsis* sp. RV163	1,6-dihydroxyphe-nazine	*T*. *brucei*^b^	19	[59]
*Actinokineospora* sp. EG49	Actinosporin A	*T*. *b*. *brucei*^b^	15	[60]
*Actinokineosporaspheciospongiae*	Fridamycin H	*T. brucei* ^b^	7.18 ^e^ 3.35 ^f^	[61]
**Cyanobateria**				
*Symploca* sp.	Symplocamide A	*L*. *donovani*^d^ *T*. *cruzi*^c^	>9.5 >9.5	[62]
*Oscillatoria* sp.	Venturamide A Venturamide B	*T. cruzi* ^c^	14.6 15.8	[63]
*Oscillatoria nigro-viridis*	Viridamine A	*L. mexicana* ^d^ *T. cruzi* ^c^	1.5 1.1	[64]
*Oscillatoria* sp.	Coibacin A	*L. donovani* ^d^	2.4	[65]
*Lyngbya majuscula*	Dragonamide A Dragonamide E Herbamide B Almiramide B Almiramide C	*L. donovani* ^d^	6.5 5.1 6 2.4 2	[66] [67,68]
*Schizothrix* sp.	Gallinamide A	*L*. *donovani*^d^	9.3	[69]
*Nostoc*78-12A	Nostocarboline	*L*. *donovani*^d^	34.3	[70]
*Okenia* sp.	Janadolide	*T*. *brucei*^b^ *T*. *b. rhodesiense*^b^ *T. cruzi*^c^	0.047 91.3 69.3	[71,73]
**Firmicutes**				
*Bacillus pumilus*	3-hydroxyacetyl-indole *N*-acetyl-β-oxotrypta-mine 3-formylindole	*T*. *cruzi*^c^	20.6 19.4 27	[74]
**Gammaproteobacteria**				
*Pseudomonas* sp.	Pseudopyronine A Pseudopyronine B	*L*. *donovani*^d^	9.8 4.65	[75]
**Fungi**				
**Ascomycetes**				
*Nigrospora sphaerica*	Aphidicolin	*L*. *donovani*^a^ *L*. *infantum*^a^ *L*. *enriettii*^a^ *L*. *major*^a^ *L*. *donovani*^d^	157 40.8 90.4 55.4 35	[76,77]
*Hypocrella bambusae*	Hypocrellin A Hypocrellin B	*L*. *donovani*^a^	0.5 24	[78]
*Penicillium janthinellum*	Citrinin	*L*. *mexicana*^a^	160 ^e,l^	[80]
*Alternaria* sp.	Altenusin	*T*. *cruzi*^g^	4.3	[82]
*Alternaria* sp. (DC401)	3,6a,9,10-pentahydroxy-7,8-epoxy-4-oxo-4,5,6,6a,6b,7,8,9-octahydro-perylene 3,6,6a,7,10-pentahdroxy-4,9-dioxo-4,5,6,6a,6b,7,8,9-octahydro-perylene	*L*. *donovani*^a^	7 12	[83]
*Cochliobolus* sp.	Cochlioquinone A Isocochlioquinone A	*L*. *amazonensis*^d^	1.7 4.1	[84]
*Edenia* sp.	Preussomerin EG1 Palmarumycin CP_17_ Palmarumycin CP_18_	*L*. *donovani*^c,d^	0.12 ^d^ 1.34 ^d^ 0.62 ^d^ 23.5 ^c^	[85,86]
*Ascochyta salicorniae*	Ascosalipyrrolidinone A 2,3-dihydro-2-hydroxy-2,4-dimethyl-5-trans-propenylfuran-3-one	*T*. *cruzi*^a^ *T*. *b*. *rhodesiense*^b^	2.6 ^h^ 70.1 ^h^ 535 ^h^ 178 ^h^	[87]
*Diaporthe phaseolorum*-92C	18-des-hydroxy cytochalasin H	*L*. *amazonensis*^a^	19.2	[88]
*Fusarium* sp. KF611679	Beauvericin	*T*. *cruz*i ^c^	2.43	[89]
*Nectria pseudotrichia*	10-acetyl trichoderonic acid A 6′-acetoxy-piliformic acid Hydroheptelidic acid	*L*. *braziliensis*^c^	21.4 28.3 24.8	[90]
*Talaromycespurpurogenus* H4	Austin	*T*. *cruzi*^i^	73.1	[91]
*Acremonium egyptiacum*	Ascofuranone	*T. b*. *brucei*^j^	0.00013	[93]
*Penicillium sp.*	Mycophenolic acid	*T. brucei* ^k^ *T. brucei* ^b^ *T. cruzi* ^c^	0.021 0.51 1.6	[99,100]
*Penicillum citrinum*	Mevastatin	*L. donovani* ^a,c^	23.8 ^a^ 7.5 ^c^	[102]
*Chaetomium* sp.	Chaetoxanthone A Chaetoxanthone B Chaetoxanthone C	*T*. *b*. *rhodesiense*^b^ *L*. *donovani*^c^ *T*. *cruzi*^c^ *L*. *donovani*^c^	12.6 9.6 3.83 8	[103]
*Aspergillus oryzae*	Kojic acid	*L*. *amazonensis*^a,c^	239 ^a^ 193 ^c^	[106]
*Geosmithia langdonii*	2,5-dihydroxybenzaldehyde	*L*. *donovani*^a^	3.3	[108]
*Eurotium repens*	Auroglaucin 2-(20,3-epoxy-10,30-heptadienyl)-6-hydroxy-5-(3-methyl-2-butenyl)benzaldehyde	*L*. *donovani*^a^	25 20.7	[111]
*Clonostachys* sp.	IB-01212	*L*. *donovani*^a^ *L*. *pifanoi*^c^	10.5 7.1	[112]
*Paecilomyces* sp. FKI-3573	Pyrenocines A Pyrenocine B Pyrenocine I Citreoviridin	*T*. *b*. *brucei*^b^	0.57 3.35 8.56 1.2	[113]
*Paecilomyces* sp. 7A22	Harzialactone A	*L*. *amazonensis*^a,c^	27.3 ^a^	[114]
			94.6 ^c^	
**Basidiomycetes**				
*Phanerochaete* sp. H2	Austin	*T*. *cruzi*^i^	73.1	[91]

^a^ promastigotes; ^b^ trypomastigotes; ^c^ intracellular amastigotes; ^d^ axenic amastigotes; ^e^ IC_50_ (48 h); ^f^ IC_50_ (72 h); ^g^ trypanothione reductase; ^h^ minimum inhibitory concentration; ^i^ epimastiogtes; ^j^ ubiquinol oxidase; ^k^ inosine 5′monophosphate dehydrogenase; ^l^ 100% activity.
